# A Global Regulation Inducing the Shape of Growing Folded Leaves

**DOI:** 10.1371/journal.pone.0007968

**Published:** 2009-11-23

**Authors:** Etienne Couturier, Sylvain Courrech du Pont, Stéphane Douady

**Affiliations:** Laboratoire MSC, Matière et Systèmes Complexes, UMR 7057, CNRS & Université Paris-Diderot, Paris, France; University of Hull, United Kingdom

## Abstract

Shape is one of the important characteristics for the structures observed in living organisms. Whereas biologists have proposed models where the shape is controlled on a molecular level [1], physicists, following Turing [2] and d'Arcy Thomson [3], have developed theories where patterns arise spontaneously [4]. Here, we propose that volume constraints restrict the possible shapes of leaves. Focusing on palmate leaves (with lobes), the central observation is that developing leaves first grow folded inside a bud, limited by the previous and subsequent leaves. We show that the lobe perimeters end at the border of this small volume. This induces a direct relationship between the way it was folded and the final unfolded shape of the leaf. These dependencies can be approximated as simple geometrical relationships that we confirm on both folded embryonic and unfolded mature leaves. We find that independent of their position in the phylogenetic tree, these relationships work for folded species, but do not work for non-folded species. This global regulation for the leaf growth could come from a mechanical steric constraint. Such steric regulation should be more general and considered as a new simple means of global regulation.

## Introduction

Leaves fascinate for their shape diversity. They can be simple, with lobes (palmate), with leaflets (compound), or dissected with holes. On one single plant, the leaf shape varies, sometimes strongly (heterophilly). Until now, botanists have proposed two mechanisms to explain leaf forms. The first mechanism is based on localised enhancements and reductions of growth of the free margin of the embryonic leaf [5]–[6], which create the peaks and the valleys of the leaf border [7]–[8]. The second mechanism is the death of patches of cells (programmed cell death, PCD) that forms perforations in the leaf during the lamina development. When perforations are positioned near the leaf contour, the marginal tissue eventually breaks, as in *Philodendron Monstrosa* (Araceae, monocotyledon), resulting in a deeply dissected blade (pinnatisect) [9]. A particular case has been described for the dissected shape of palm leaves (Arecaceae, monocotyledons). The leaf first develops with many folds, along which PCD eventually takes place, creating cuts [10].

These two mechanisms are general so that they can be tuned to reproduce the final shape of any leaf. They conceptually apply for a flat leaf during its expansion and do not take into account the actual geometry of the growing leaf inside the bud. Only the last case takes into account this geometry, through the folds. Folds have been recently highlighted for their mechanical importance in thin sheets [11]–[12] and are ascribed to play a role in the expansion of hornbeam leaves [13]. Focusing on palmate leaves, we found that most of them are first growing folded inside a bud. We show here that these folds have a direct influence on the final shape of the leaf.

**Figure 1:**
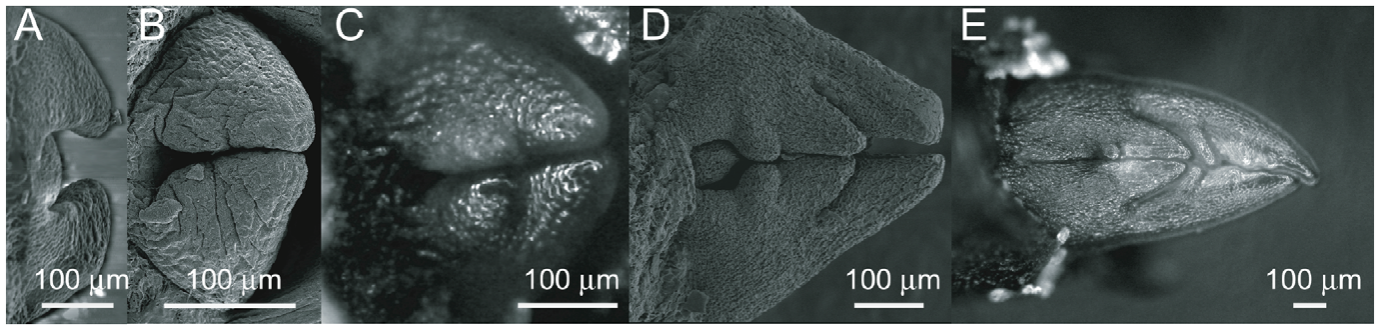
Developing embryonic leaves of *Acer pseudoplatanus*. For such opposite decussate phillotaxy, a pair of symmetric leaves develop simultaneously. The primordia first expand over the stem apex (A) and then the two symmetric leaves meet (B), limiting each other in their future growth (C–E): a first lateral fold has appeared between the central and the lateral veins (C), a second one (D), and a secondary vein on the central one (E). Pictures A, B and D are MEB pictures courtesy of Isabel Le Disquet from IFR 83 of UPMC-Paris 6. Pictures C and E are optical microscope pictures.

Leaves are highly organised botanical elements. They originate from small groups of cells (primordia) protruding around the shoot apex (fig. 1A). From the beginning they present a fundamental asymmetry: the side turned toward the stem axis (adaxial) will become the smooth and shiny upper side of the leaf turned toward the light; the other side, turned toward outside (abaxial), present hairs and veins protruding and will become the lower side of the leaf.

**Figure 2:**
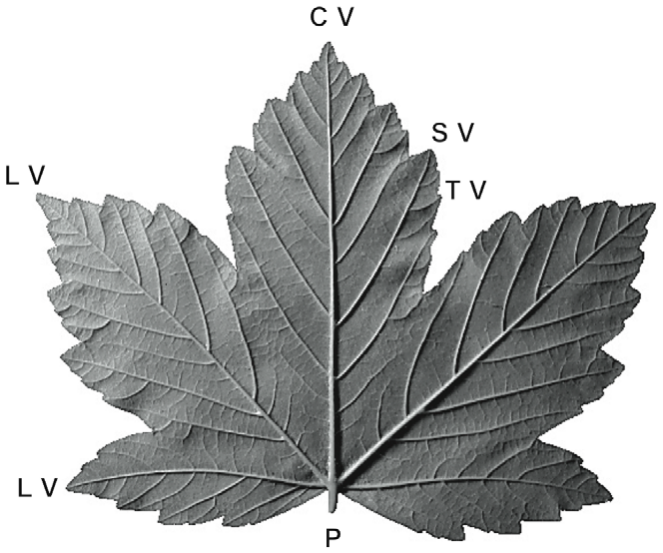
A mature leaf of *Acer pseudoplatanus*. The largest lobe corresponds to the central vein (CV), while the lateral lobes develop around other major lateral veins (LV), radiating from the end of the petiole (P). Secondary lobes correspond to the end tip of some secondary veins (SV). There can be rare and small third order lobes around some end tip of third order veins (TV).

Once the leaf has grown, it can be noticed that the final vein pattern is organised and hierarchical. Along a central vein runs majors lateral veins, radiating from the petiole, at the base of the leaf. The secondary veins branches out from them, and so on and so forth. It is also noticeable that in palmate leaves the vein hierarchy is related to the leaf shape (see figure 2). Each lobe corresponds to a major vein ending at its tip. Lobes and veins have the same hierarchy: the central vein (CV) corresponds to the largest lobe, with lateral lobes corresponding to other main lateral veins (LV), secondary lobes corresponding to secondary veins (SV), up to a rare third order lobes (TV).

In buds, leaves grow in a limited space defined by previous and following leaves. To keep on expanding its future lamina within this confined space, leaves either enroll (convolute, revolute or involute), or fold (plicate and palmate leaves) [14]–[15]. This fact has been noticed early, but overlooked since the XIXth century.

## Results

**Figure 3:**
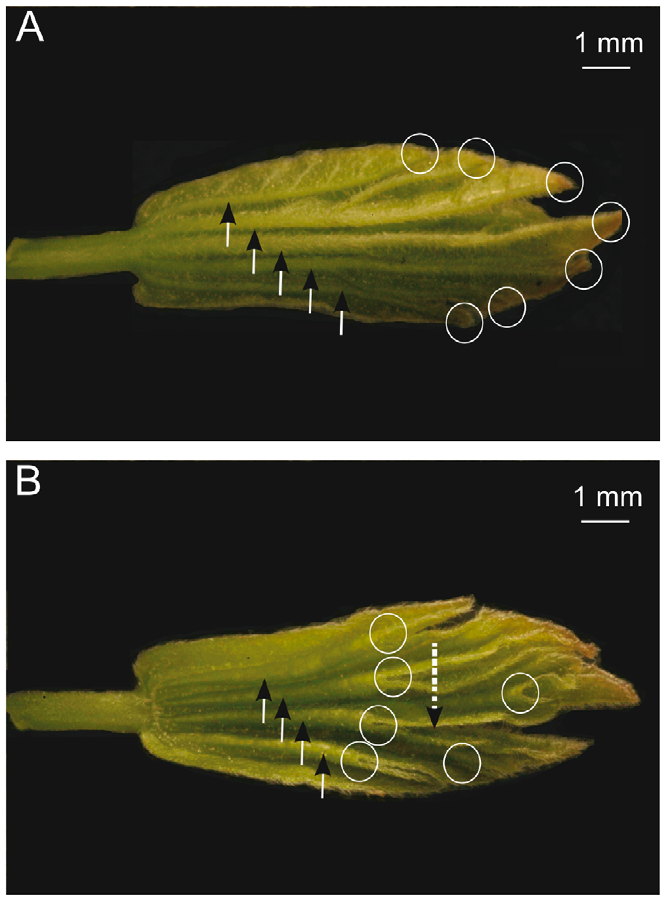
Folded immature leaf extracted from a bud of *Acer campestre*. A: The abaxial side shows the anticlinal folds (arrows) running along veins and ending at peaks (circles). B: The adaxial side shows the synclinal folds (arrows) running along “anti-veins” and ending at sinuses (circles). Peaks and sinuses stand in the contact plane of the pair of leaves (figure 1E), but while peaks are at the extreme of this contact surface, sinuses are inside.

Our first remark is that in palmate leaves, folds are not irregular but strictly follow the leaf organisation and hierachy (see figure 3). Following the leaf asymmetry, the folds showing the abaxial (lower) epidermis outside (anticlinal folds) are very different than the opposite ones, showing the adaxial (upper) epidermis outside (synclinal folds). The anticlinal folds coincide with the main veins and follow their hierarchy (figure 3A). This relation is inclusive: an anticlinal fold always corresponds to a main vein while many veins do not correspond to any fold. On the contrary, synclinal folds do not correspond to any major vein and are rather crossed only by the smallest ones (figure 3B). Thus we call them “anti-veins”.

**Figure 4:**
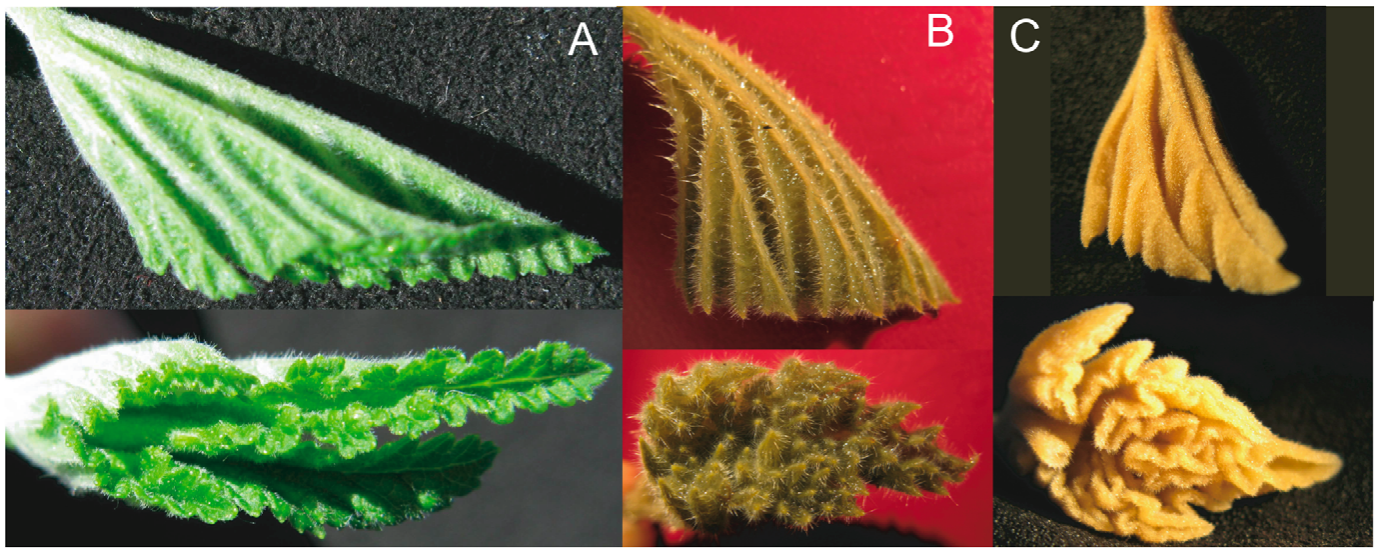
Side and front views of Kirigami leaves. The side view shows the correspondence of anticlinal folds with the main veins, the front view shows the adaxial contact plane, where the leaf perimeter is located. A: *Ribes nigrum* (Saxifragales), B: *Pelargonium cuculatum* (Geraniales), C: *Malva sylvestris* (Malvales).

Our second and main observation is that the whole perimeter of folded leaves growing inside a bud is located at a particular place. For Palmate leaves it is located on the adaxial side, at the border of the folding volume, toward the central axis of the stem (see figure 4). As the leaf margin links the end of synclines to the end of anticlines, this last property induces strong relationships between the shape of the leaf and the way it was folded inside the bud. This is similar to Kirigami (‘cut-paper’ in Japanese), where a piece of paper can take any shape by folding it in a particular way, and then cutting it on one side [16].

**Figure 5:**
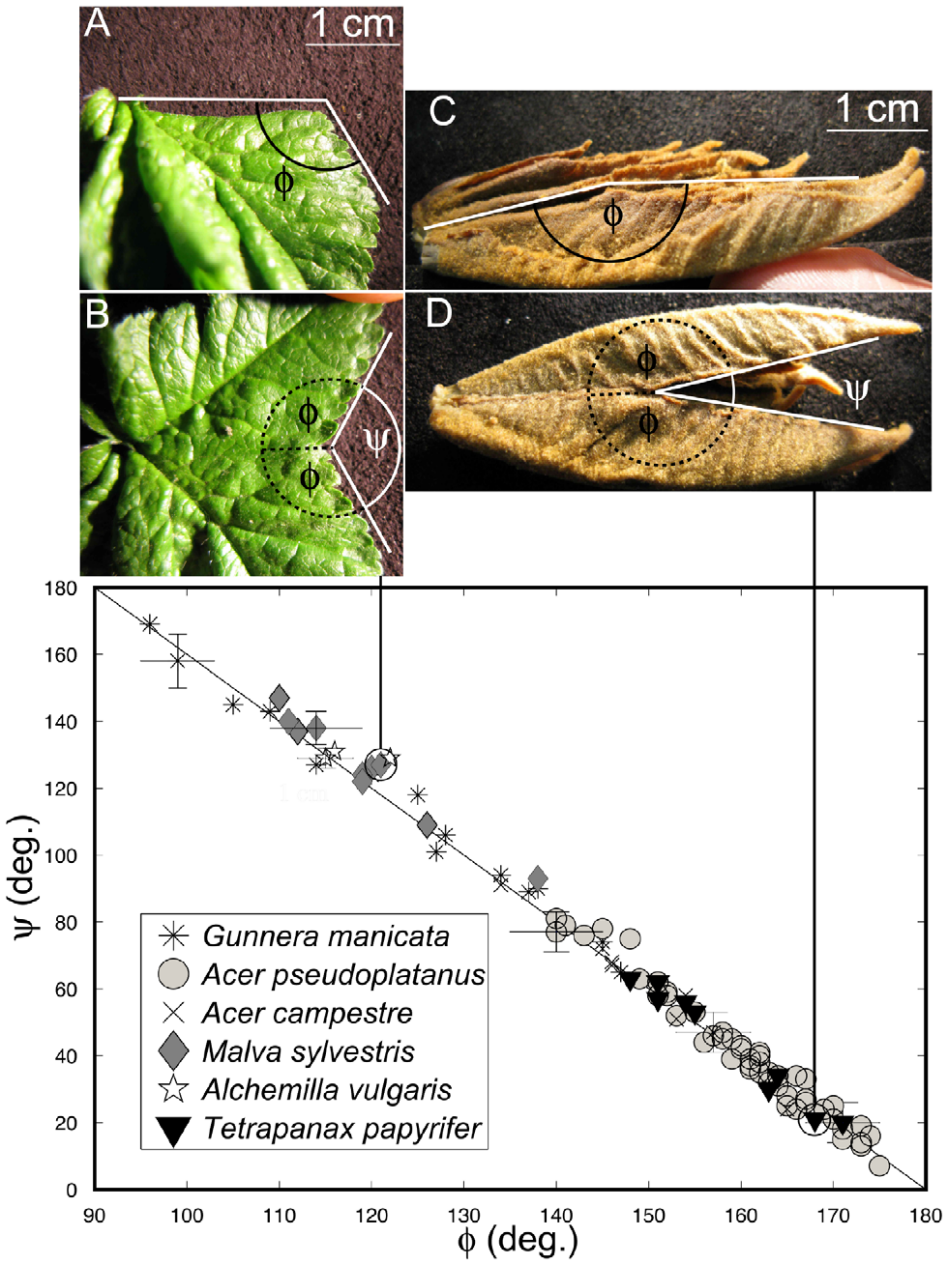
Local relationship between synclinal folds and sinuses on immature leaves before their expansion outside the bud. Synclinal fold angles φ are measured from side views on folded leaves extracted from buds (A and C). Leaves are then unfolded to measure the corresponding sinus opening angle ψ from a top view (turning the back in b, and turning the top in d). The fold, setting the sinus whose contours locally superimpose, is the symmetry axis of the sinus. If the leaf is a folded flat surface, this symmetry simply makes ψ  =  360° − 2 φ, as sketch in B and D. This relation (line) is checked for many folds on different leaves and species. The fact that it works shows that even if the leaves grow folded in the bud (figure 3), they are already flat. The plot also shows the large range of angle variation within one species and for all the species. Pictures are two extreme cases: when the fold angle become close to 90° the valley disappears (Malva, left), while when it becomes close to 180°, the valley becomes a simple cut (Tetrapanax, right). The studied palmate leaf species belong to different order of eudicots. Following the APG II classification *Acer pseudoplatanus* and *Acer campestre* (Sapindales), *Malva sylvestri*s and *Alchemilla vulgaris* (Malvales) are Rosids, *Tetrapanax papyriferum* (Apiales) is an Asterid and *Gunnera manicata* is a Gunnerale.

The first consequence is that with these particular folds, and with the leaf perimeter turned adaxially, anticlines set peaks (or lobes) and synclines set valleys (or sinuses) of the unfolded leaf. As anticlines correspond to main veins, this results in the lobes surrounding the main veins. Even more, for the folded leaf, the two sides of the perimeter superimpose around a fold. The peak and sinus must then be symmetric around the fold. This seems natural for lobes, but Figure 5 shows this local property for sinuses on immature leaves extracted from buds of various species.

**Figure 6:**
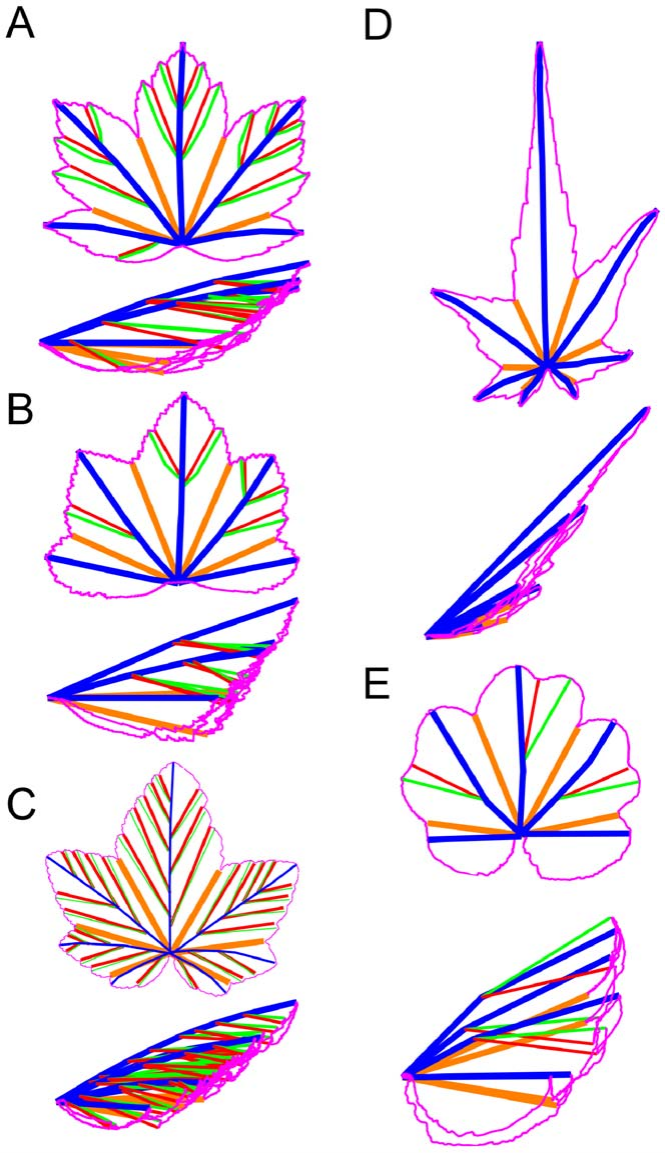
Mature leaves of different species, numerically folded back. The leaf contour is pink. Primary and secondary veins are respectively blue and green. Primary and secondary anti-veins are yellow and red respectively. Only veins ending at peaks are represented and stand for anticlinal folds along segments linking two consecutive branching points or a branching point to a peak. Synclinal folds run along segments (anti-veins) linking a sinus to the branching point of the two surrounding veins. The thickness of the leaf is not taken into account and the leaf is folded back onto a plane, holding the angles to the best (see material and methods, figure 13). A: *Acer pseudoplatanus*, B: *Malva sylvestris*, C: *Ribes nigrum*, D: *Sida hermaphrodita*, E: *Gunnera manicata*. Following the APG II classification, these species belong to different orders of core eudicots: *Acer pseudoplatanus* (Sapindales), *Sida hermaphrodita* and *Malva sylvestris* (Malvales) are Rosids, *Gunnera manicata* is a Gunnerale and *Ribes nigrum* is a Saxifragale.

**Figure 7:**
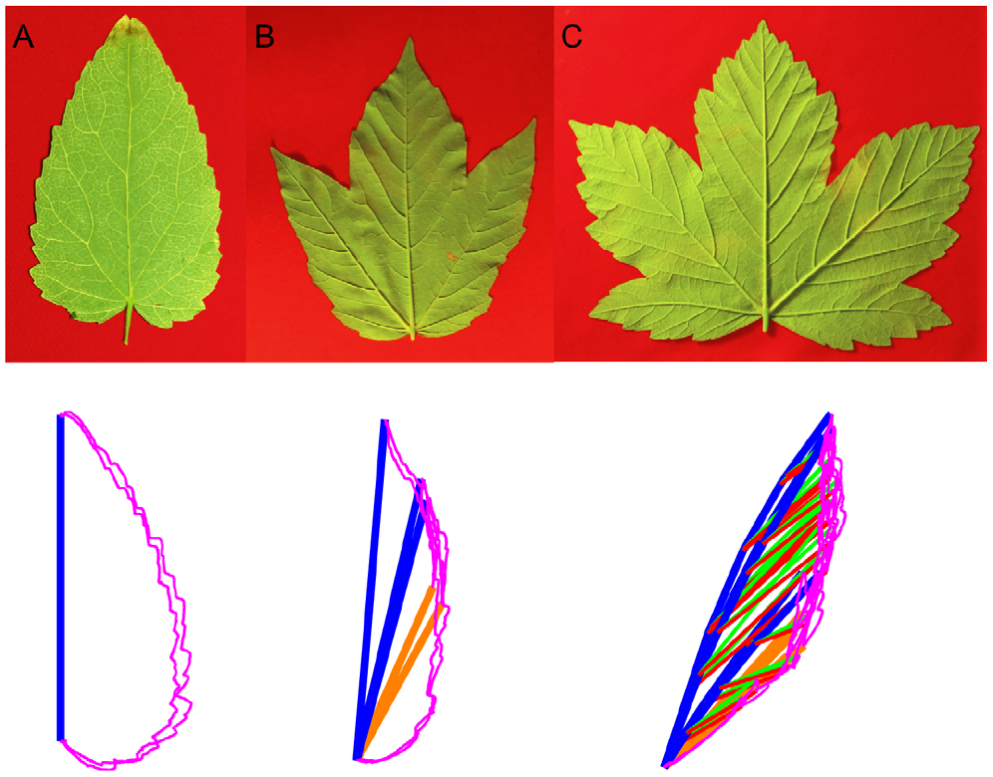
*Acer pseudoplatanus* leaves: pictures and numerical folding (same representation as figure 6). A: One lobe leaf, with only one anticlinal fold. B: Three lobe leaf, with two lateral anticlinal and three synclinal folds. C: Five lobe leaf, with four lateral anticlinal and five synclinal folds, and several secondary folds (as in figure 6A). The secondary folding is necessary here to obtain a refolding of the contour on a simple curve (see figure 13). One lobe leaves are found on new sprouts.

This Kirigami property not only induces local geometric relationships in immature leaves, but also global ones that are kept in the mature leaves, determining their final shape. Using the relation between the folds and the main veins (first and second order), mature leaves can be folded back like we expect they were when growing in the bud. Figure 6 shows various mature leaves folded this way. One can see that the whole leaf perimeter collapses onto a simple curve, even for asymmetric leaves. The different leaf shapes can then be ascribed essentially to the variation of angles between the veins and to the different curves delimiting the folded leaf. Within a single species, the change in the number of lobes can just be ascribed to a change in the number of folds (see figure 7).

**Figure 8:**
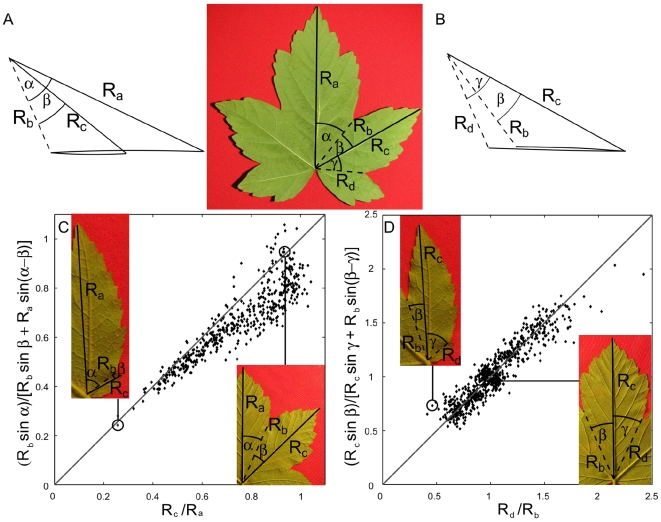
Geometric relationships between two successive lobes and sinuses coming from the Kirigami property. A: Two consecutive primary lobes have veins of lengths R_a_ and R_c_. They are respectively making an angle α and β with the anti-vein, of length R_b_, between them. B: The vein of length R_c_ is surrounded by two anti-veins of lengths R_b_ and R_d_. These are respectively making an angle β and γ with the vein. C: According to A where the folded “leaf” contour is the same for both sides of the fold, it is possible to compute R_c_ from R_a_, R_b_, α and β (see formula and figure 14 in methods). For β growing from 0 to α, R_c_ simply run along the perimeter, growing from R_b_ (left inset) to R_a_ (right insert). To compare leaf of different sizes, we plot R_c_ divided by R_a_, compared with the Kirigami prediction. D: Similarly, for tow anti-veins surrounding a vein, when β grow from 0 to γ, R_b_ decrease from R_c_ (right inset) to to R_d_ (right inset). As the anti-veins were not ordered by size (contrary to the veins), the figure is symmetric. Points represent 121 sycamore leaves. For each leaf, all pair of consecutive primary veins or anti-veins (four for the leaf presented) have been measured.

**Figure 9:**
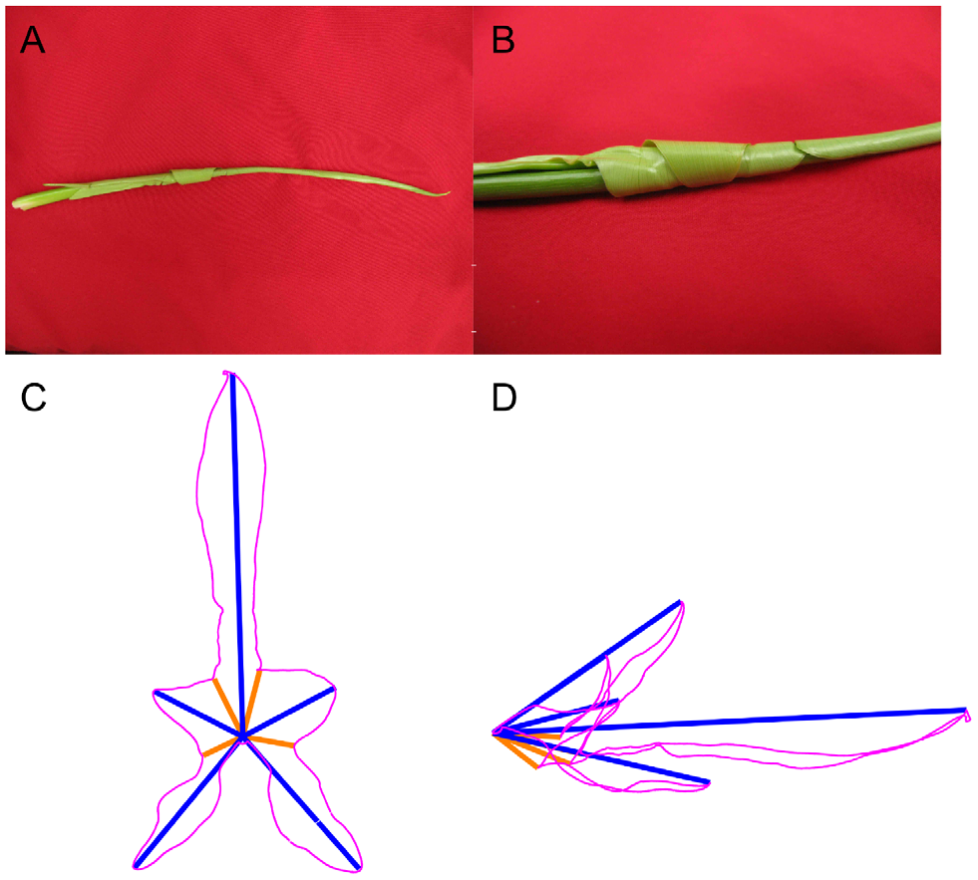
Example of an enrolled leaf: the *Philodendron bipenifolium* (Araceae). A: A juvenil *Philodendron bipenifolium* leaf. B: Detail of the enrolled leaf. The leaf does not grow folded but enrolled; it is an involute leaf. C: A mature *Philodendron bipenifolium* leaf. D: The same leaf numerically folded. Like for folding of figure 6, the thickness of the leaf is not taken into account (see figure 13 and material and methods for a detailed explanation). As the leaf does not grow folded, it does not obey the Kirigami property and is not foldable with its contour lying on a simple curve.

**Figure 10:**
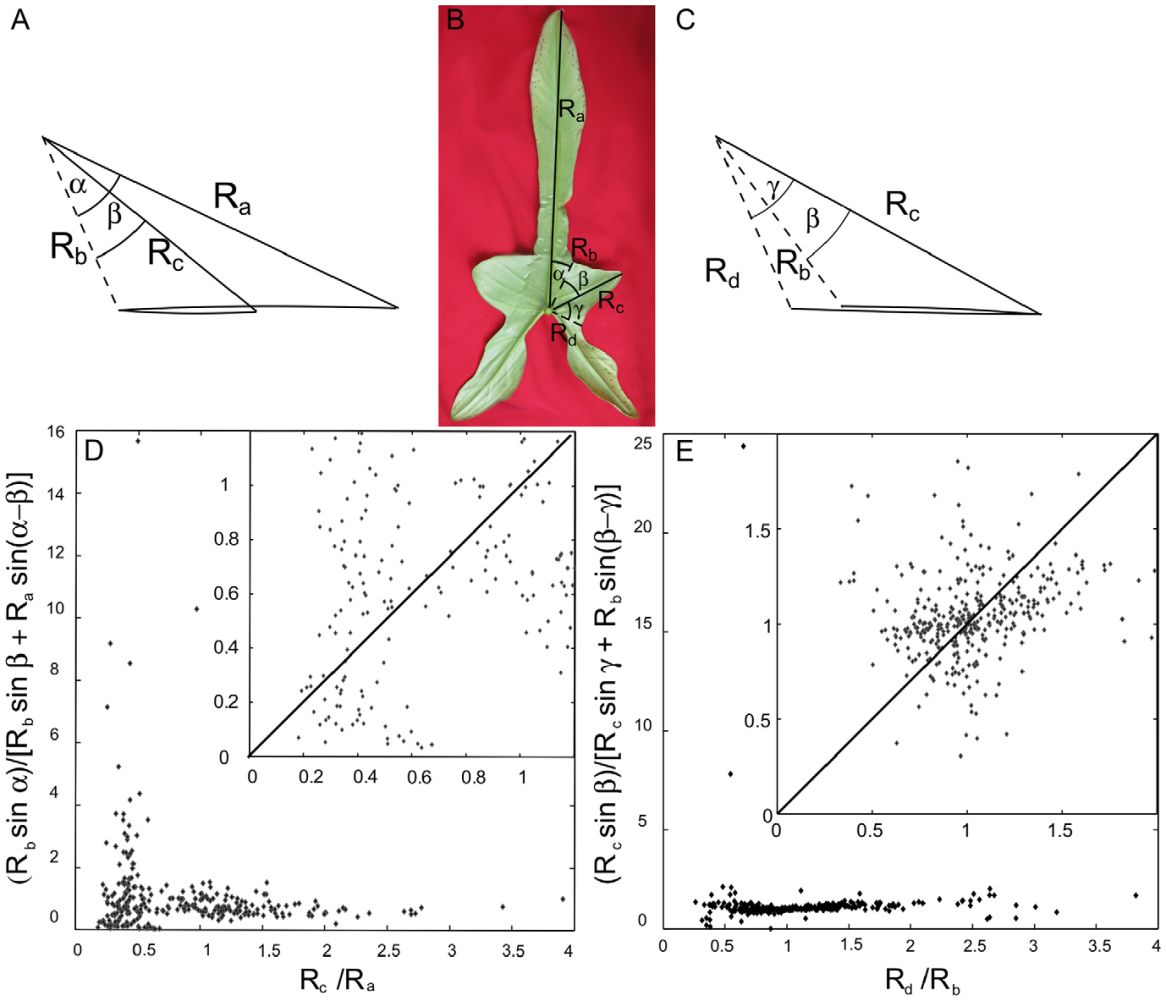
Geometric relationships between two successive lobes and sinuses coming from the Kirigami property, for the enrolled *Philodendron bipenifolium* leaf. Same notations and plots as in figure 8. A: Two consecutive primary lobes have veins of lengths R_a_ and R_c_. They are respectively making an angle α and β with the anti-vein, of length R_b_, between them. B: The vein of length R_c_ is surrounded by two anti-veins of lengths R_b_ and R_d_. These are respectively making angle an β and γ with the vein. As in figure 8, the predictions of the Kirigami property are compared to the measured values for veins (C) and for anti-veins (D). Points represent 85 *Philodendron bipenifolium* leaves. For each leaf, all pair of consecutive primary veins or anti-veins (four for the presented leaf) have been measured. One observes that points are scattered in both figures. This figure, together with the previous one, shows that the Kirigami property does not appear for any shape.

This property can be translated into quantitative relationships between the length of veins and anti-veins and the angles between them. Sharing the same perimeter once folded induce that the lobes and sinuses follow some proportionality. These relationships, restraining the range of possible shapes, are shown in the case of sycamore leaves in figure 8. As well as in figures 5 and 6, these relationships hold for palmate species widely separated on the evolutionary scale (APG II classification)[17], while more closely related species with leaves that do not grow folded inside the bud show no trace of such organization. This is shown in figures 9 and 10, for Philodendron bipenifolium (Araceae) leaves. Even though it presents lobes, it grows enrolled inside its protective envelope (figures 9A, 9B). When the leaf contour is folded following the veins and anti-veins (fig. 8c), the contour does not coincide (figure 9D). When the geometrical prediction is checked, the points are scattered on the whole plane (figure 10).

**Figure 11:**
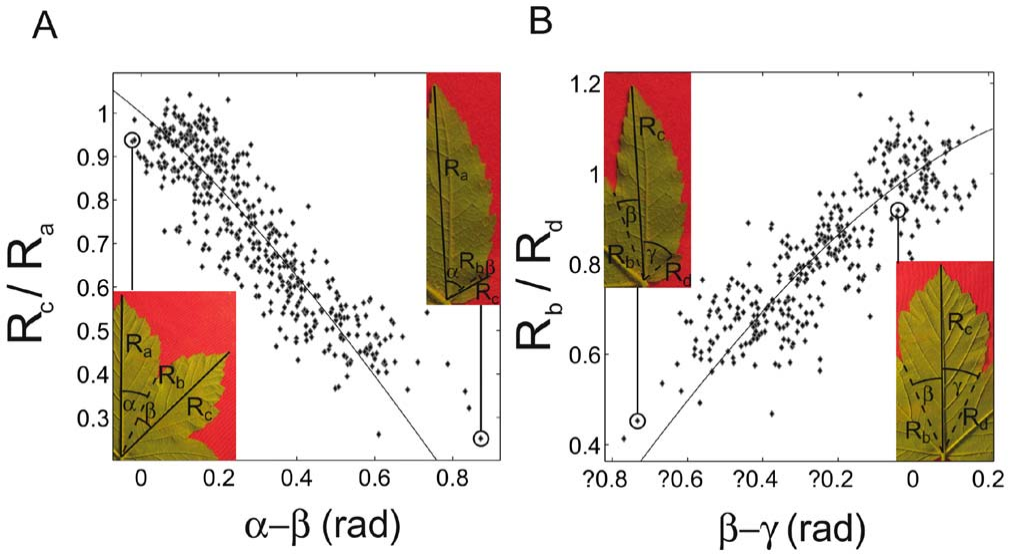
Simplified relationship on two successive lobes and sinuses that can be used to recognize the Kirigami property. As in figure 8, the graphs express the relation between the folds length and the angles. A: Length ratio (R_a_/R_c_) of two consecutive main veins in function of the difference (α–β) between the angles there are making with the anti-vein. The smaller the angle, the smaller the vein. If the anti-vein (sinus) is at the middle between the two veins, then the two veins have the same length (ratio 1, right inset). On the contrary, if the angle for the second lobe becomes small, then the length of the second lobe should be smaller, eventually becoming equal to the length of the anti-vein or sinus (left inset). B: Length ratio (R_b_/R_d_) of two consecutive main anti-veins in function of the difference (β–γ) between the angles with the vein. Similarly, the relation express that the smaller the angle, the longer the anti-vein. When the angles are equal the two anti-veins have the same length (right inset), while if one angle becomes smaller, the corresponding anti-vein becomes longer, eventually becoming equal to the vein (left inset). Contrary to figure 8, these relations are approximate, scattering the points. They are exact only if the opening angle of the lobes is constant, which is a first approximation, but in practice mixes different relationships for each lobe opening angle. However, they are simple way to judge of the Kirigami property, judging by the eye the ratio of length and angle difference, that the anti-veins and veins are axes of symmetry of the contour. For instance they are clearly wrong for figure 10B (α is smaller than β, but R_a_ is much longer than R_c_).

The previous geometrical relationship can be intuitively translated, allowing to recognize at first sight a Kirigami leaf (figure 11). The fact that the perimeter was folded back on the same curve means, for two consecutive lobes, that the longest is the one whose vein makes the biggest angle with the separating anti-vein fold (figure 11A). Similarly, for two sinuses around a middle vein, the smaller of two sinuses makes a bigger angle with the middle vein (figure 11B).

## Discussion

These observed geometrical relationships relate directly to the way leaves grow folded. These folds develop successively around the main veins. At the same time, a leaf lobe grows around each main vein. From a genetic point of view, each lobe can be seen as a new independent primordium. The same KNOX gene is expressed both at the place of appearance of a primordium and at the appearance of a new lobe [18]. As well the same CUC gene is expressed both at the separation between the primordium and the meristem, and in between the new lobes [19]. But what correlates the development of these lobes so that the final result fits this Kirigami property, bringing the lobe perimeters at the same volume border? The answer could be contact regulation, as mechanical contacts are known to regulate the global expansion and growth of plants [20]–[21].

Based on many observations of dissected buds at different stages (figure 1), we can write the following scenario for the development of the leaf inside the bud. The leaf primordium first develops, with a central vein and a flat lamina (figure 1A). It extends over the meristem apex, until it reaches the other leaves (figure 1B). The fact that it stops and will never overpass this limit indicates a first contact regulation of the growth. Later on, secondary veins being already there [22], folds appear. We think that these main veins have an active role in the formation of the folds: they rotate the lamina around them, creating the synclinal folds. This active folding pushes the rest of the developing lamina toward the inside, inducing rounded anticlinal folds (figure 1C). The lobes develop around these folded main veins, but the developing lamina stops its growth when reaching the volume border, indicating the again a contact regulation of the growth (figure 1D).

Thus the development of the lobes depends on the folding around the main veins. This active process can be triggered by the lateral mechanical constraint, but not necessarily, as neo-formed leaves can develop outside the bud and still fold themselves. Further phenomena, like PCD in the case of palm leaves, or inhomogeneous growth in the case of oaks, can also intervene to eventually shape the final leaf form once it is outside the constraining volume of the bud.

Even though growing folded in the bud, it is remarkable that the embryonic leaf can be unfolded nearly as flat as the final leaf (figure 5). This already proves a strong and permanent regulation to keep the leaf lamina flat locally [1]. But to have an overall flat surface, the folds, where this regulation mechanism cannot apply, have also to be considered. If the folds are straight, as elongating veins tend to be, then the overall surface can be flat. A problem remains around the synclinal folds, that are not restricted *a priori* to be straight. Indeed some leaves exhibit curved synclinal folds, and then a characteristic half-saddle unflatenable shape at their sinus.

**Figure 12:**
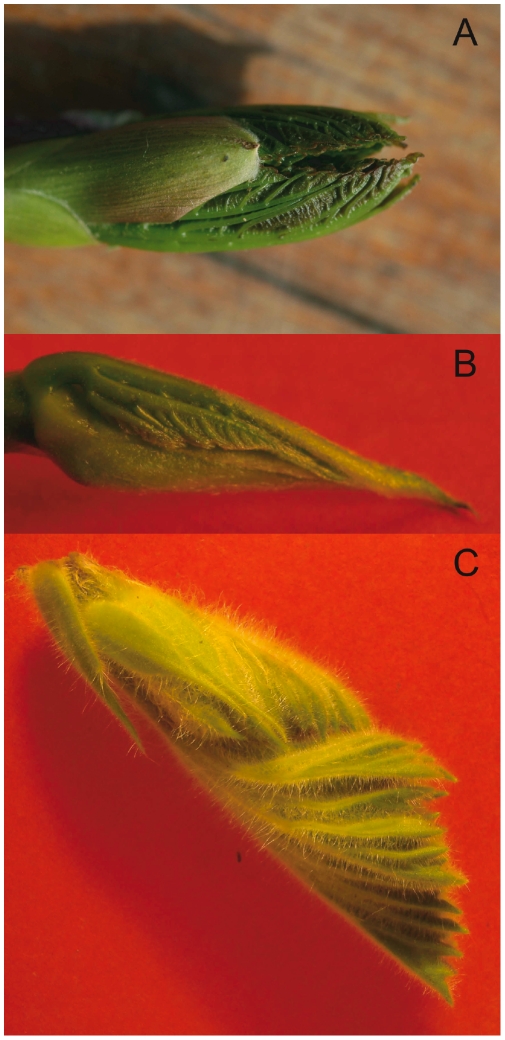
Dissected Kirigami buds showing the packed folded leaves. One leaf can be limited by an other leaf of the same age (opposite decussate phyllotaxy, A, while there are also two other pairs of leaves, older and younger, delimiting the volume); or leaves of different ages (spiral phyllotaxy), either protected by an envelope, B, or not, C. A: *Acer pseudoplatanus* (Sapindales), B: *Murus platanifolium* (Rosales), C: *Pelargonium cuculatum* (Geraniales). In all cases, the leaf fit the available volume, and no space is left free in the bud.

The evolutionary interest of such folding mechanisms could be not to regulate the final shape of the leaf, but to protect the very fragile immature leaves. A good way to protect them is to grow them inside buds with protecting scales. In the limited volume of the bud, one way to develop the largest surface, ready to catch light, is to grow folded. We found that the leaves grow with their own internal dynamics, create folds, and stop when reaching the constraining surface. This overall growth regulation is a simple way to ensure that the whole volume is evenly occupied, and that no space is lost (see figure 12). Indeed, buds are always completely full with no free space remaining. The asymmetric development of the folds (figure 1), with the fragile lamina pushed toward the more protected adaxial side, and the more robust veins, often with hairs, covering the whole abaxial side, could also be evolutionary interesting. Not only the bud scales, but also this asymmetrical folding geometry, protect the leaf from cold, dryness, and the numerous small predators (insects). The leaf shape, with its lobes, could just be a secondary consequence of the interplay of the optimization and protective mechanisms, achieved through the folding and the growth contact regulation. This explains the observation of the predominance of palmate leaves in cold-temperate regions [23], where this protection is most needed. The variations in duration and volume development before outside expansion could finally explain the continuous variation of shapes and the number of lobes.

This global mechanical regulation of the shape could happen in leaves as their shapes are, like phyllotaxis [4], just the consequence of a packing problem and not essential for their reproduction success, contrary to the shape of flowers, that have to be more directly controlled [24]. The evolutionary pressure on leaves is to grow protected, as large and as soon as possible, whatever the growth conditions. It can be more efficiently achieved with the folding and global regulation that we proposed. The presence of this Kirigami property along the whole evolutionary tree also shows that is not a highly fixed and stabilized property.

### Conclusion

We are the first to reveal that the shape of palmate leaves, even though varying a lot on one branch of a tree, with lobes of different sizes, grow folded and follow this simple Kirigami property of a perimeter folding back on a simple curve. We observed this property on many species widely spread on the phylogenetic tree. Throughout our observations, on all the available species (including those from botanical gardens), we found only a few exceptions like *Philodendron bipenifolium*. This property of growing folded with a protected perimeter also extends to other types of leaves such as pinnate and pluri-pinnate leaves.

The kirigami property leads to geometric consequences, that we checked on young and mature leaves. This property originates from the folding of growing leaves and we insist on its importance for the final leaf shape. This folding stage and its regulation is necessary to bridge the gap between the very first primordia stages of development, which is being thoroughly studied, and the final shape of the leaves. The fact that the perimeter of these different folded lobes fall at the same border reveal a regulation process, aimed at filling perfectly the bud volume, and we propose it to be a mechanical contact regulation.

The regulation processes revealed by this leaf development study (the folding of the leaf around the veins, the mechanically sensitive leaf margin, and the overall flatness of the leaf), deserve to be more studied, and in particular their underlying molecular mechanisms. Our shape theory also points out how much the simple physical constraint of growing inside a finite volume can have an important effect, widely underestimated up to now. The theory could have implications on final shapes of organs whenever the growth is limited inside a constraining volume, such as lungs, liver or crabs legs. It finally shows how little is still known of the various shapes and their origin, not to speak of their control.

## Materials and Methods

### Materials

*Acer pseudoplatanus*, *Acer campestre*, *Malva sylvestris*, *Alchemilla Vulgaris*, *Ribes nigrum* and *Murus platanifolium* leaves were picked either in October either in June in different gardens and forest in and around Paris, France. *Philodendron bipenifolium* leaves originate from the green houses of the bontanical garden of the Serre d'Auteuil, Paris, France. *Sida hermaphrodita* Leaves come from the Botanical Garden of the Jardin des plantes (National Museum of Natural History). *Tetrapanax papyrifer* leaves come from our lab specimens, Pheonix Botanical garden in Nice, and Val Rahmeh Botanical garden in Menton. *Pelargonium cuculatum* Leaves come from the Botanical garden of Bern (Bern University), Swizerland.

Buds of *Acer pseudoplatanus* and other species were collected in late spring. Neo-formed leaves get their shape (folds) when they are bigger than pre-formed leaves which pass fall and winter in buds. It makes both the dissection and the observation of the first development stages easier.

### Scanning Electron Microscopy

The tissues were fixed within a 2% of glutaraldehyde in 0.1 M phosphate buffer solution of pH 7.4, for 7 hours. After having been rinsed with PBS they were immersed for 1 hour in a phosphate buffer solution with 1% of osmium tetroxide. They were then rinsed in water. The specimens were dehydrated through a graded alcohol series. They were dried in a critical point dryer (CPD7501 polaron) with liquid carbon dioxide. Dried specimens were coated with a gold layer about 40 nm thick with a sputtering (scancoat six sputter coater Boc Edwards). Preparations were observed with a S 260 Cambridge SEM, operating at 10 kV.

### Analyses

For the sinus opening angles, as in figure 5, the immature leaves are dissected from the buds. A photography is made of each fold, side view, and then unfold, top view. The angles are measured from the pictures.

**Figure 13:**
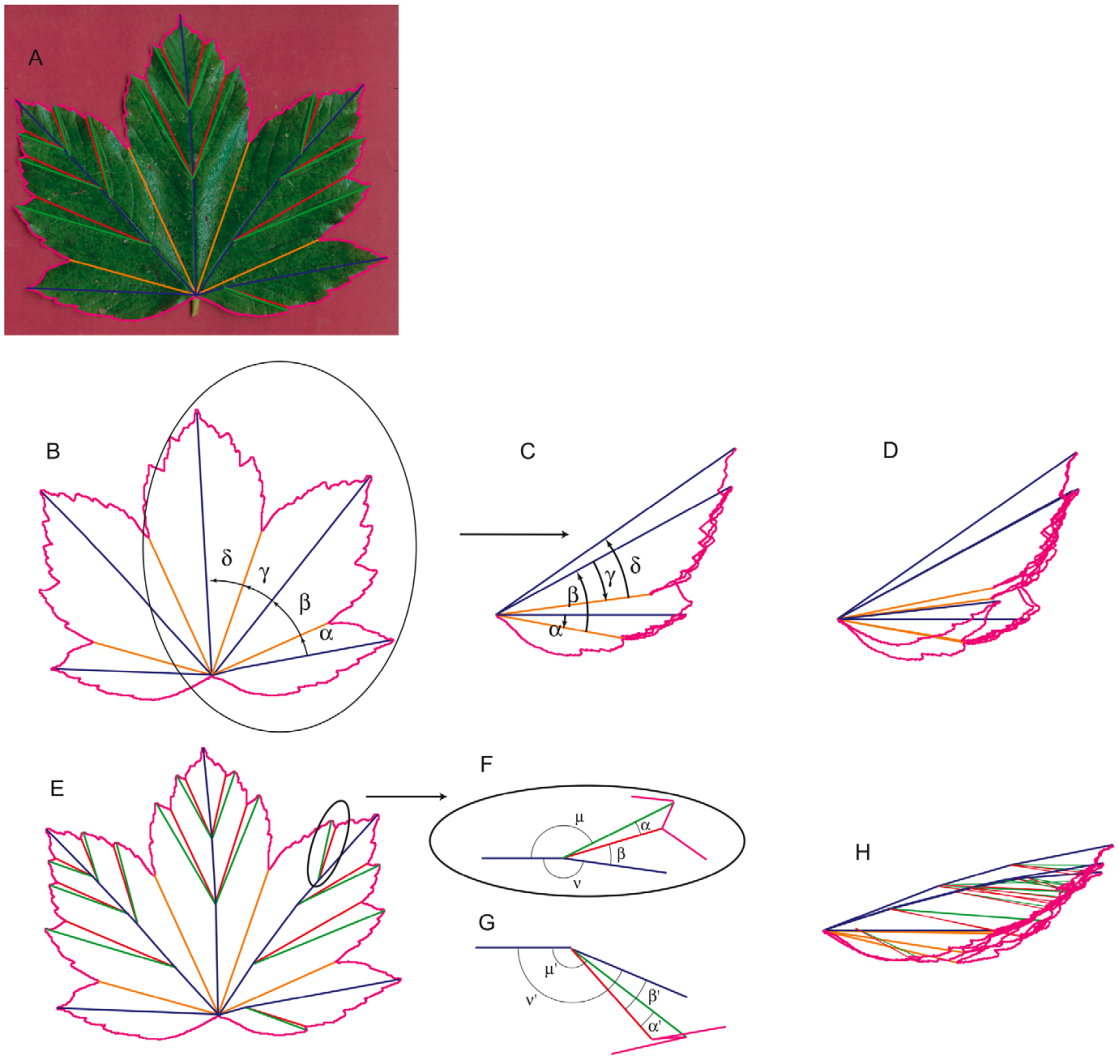
Numerical folding method. A: On a leaf, the first order vein (blue line) of a maple leaf and secondary vein (green line) are drawn. The first order anti-vein (orange line) and the second order anti-veins (red line), always going from the intersection of two vein up to the sinus between the lobes, are also drawn. The contour of the leaf (mangenta line) is numerically detected. B: The result with the contour and only the main veins and anti-veins, and the first angles between them. C: Half of the precedent sketch once refolded. α and γ are reversed, and their respective contour drawn inversed. D: The whole refolded leaf, using only its main folds. E: Sketch of the leaf with all its folds: main and secondary ones. F: Scheme of a secondary fold, unfolded, and, G: folded in a plane. The news angles are obtained as described in the text. H: The whole set of veins and anti-veins is drawn, with their respective contour, giving the completely refolded leaf.

For the numerical folding (as in figures 6, 7 and 9) and measurements (as in figures 8, 10 and 11), the unfolded mature leaf is pictured on a red background, allowing to extract numerically (by color contrast) the perimeter of the leaf (figure 13A). Then on the picture the main veins and secondary veins are drawn (by hand), keeping the numerical coordinates (see File S1). If only the main veins are used, we draw them straight from the petiole to the tip (figure 13B). Deviations from the real veins are usually small. High deviation indicates an important secondary deformation during expansion and the leaf is not used. If the secondary folds are used for numerical refolding, the veins are drawn straight from one vein intersection to the next, and finally to a lobe tip. The anticlinal fold (or anti-veins) are drawn straight from the veins intersection to the sinus (lower point) of the perimeter (it is he same for primary and secondary anti-veins: anti-veins never connect to anything else than a vein meeting point, and a sinus). For the measurements (figures 8, 10, and 11), we used only the straight main veins and anti-veins, taking their length and separation angles. For the Data and Software, see below and the File S1.

### Numerical Folding Method

Leaves are numerically folded back using the drawing of their veins (synclinal folds), contour and anti-veins (anticlinal folds, figure 13A). We first fold the main vein and anti-veins. They all join in the same point, at the end of the petiole. To refold the leaf, we just measure the angles between the successive veins and anti-veins (figure 13B), and redraw the leaf contour by inverting the sign of one angle on two, and correspondingly inverting the drawing of the contour piece. For instance, the piece of contour between the first vein and anti-vein is drawn inversed, with the vein horizontal. From the anti-vein, we then draw the next piece normally, between this anti-vein and the next vein. We then subtract the next angle, drawing the next vein/anti-vein piece inverted, add the next anti-vein/vein piece (figure 13C), and so on and so forth for the whole leaf (figure 13D).

To fold secondary folds there can be a geometrical problem. It is not always possible to keep the actual angles as resulting the folded sheet may not lie flat. The method used to numerically fold the leaves onto a plane keeps fold lengths and aims to keep at best the angle values between the folds. Finding close values is a way to project the folding into the plane (or look at it from the side), with minimal distortions. Lets consider the branching detail on figure 13E, sketched in figure 13F. When unfolded, a secondary vein is branching at a primary vein with an angle μ. The primary vein is making an angle ν at this branching point. Between these two anticlinal folds, *i.e.* the secondary and the primary veins, stands a synclinal fold that makes angles α and β with them. To these angles α, β, μ and ν for the unfolded leaf, correspond the angles α', β', μ' and ν' when the leaf is folded (figure 13G).

The sum of these angles for the unfolded leaf is of course:
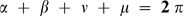



Considering the angle ν', the sketch of figure 13G is in a plane only if:
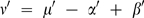
(1)


If the sum of folded angles also follows (still on a plane unfolded)
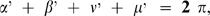
equation (1) rewrites:
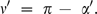



Folding the branching with keeping to the best the angle ν is then minimizing the quantity:
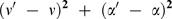
which rewrites
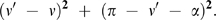



One finds the best ν′ value:
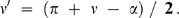



In the same way, one finds:
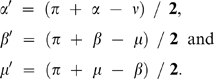



Once the angle corrected, the whole figure of folded veins and antiveins is drawn, and finally the contour is drawn for each vein-antivein segment, reverted if necessary, and with its angular position stretched or compressed if necessary, keeping the distance to the fold center (figure 13H). Usual corrections from the actual angles are around few degrees. For instance, in figure 6, the corrections are around 1.5°.

### Data and Software

Data from the figures and software (in Matlab) are given as File S1. The data are the direct measurements of the positions of the petiole and main peaks and sinuses in many leaves, corresponding to figures 8, 10 and 11, together with the software allowing to extract the lengths and angles from them, and plot them as in the figures (folders: figure 8 and 10 – data; figure 11 – data). The Folding Software allows, once the contour of the image is obtained, and the information of the main and secondary veins and anti-veins obtained (via a sub-software), to fold back the contour. The cases form figures 6 and 9, with the original pictures, are provided as examples (folder: figure 6 and 9 - Folding and data). Short manuals are included.

### Formula

**Figure 14:**
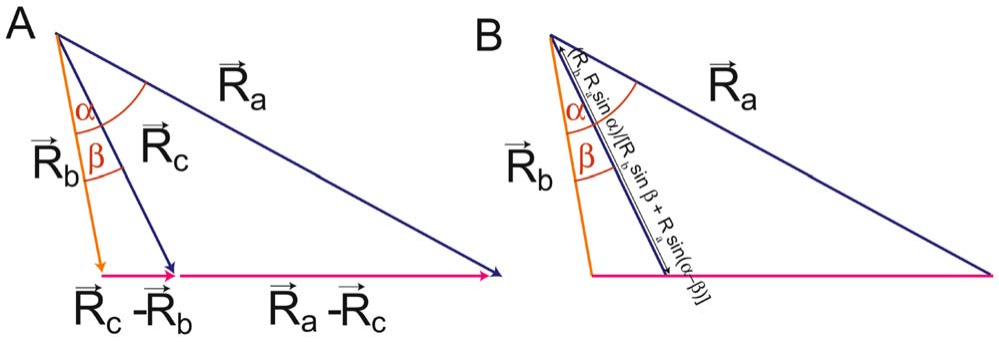
Derivation of a formula. A: If the tips of R_a_, R_b_, R_c_ align then the two vectors (R_c_ –R_b_) and (R_a_ –R_c_) are collinear. Their cross product is null. B: For a given set of α, β, R_a_, R_b_, with this property, one can deduce R_c_.

Taking the sketch of figure 8A or figure 14, the condition that contour superimpose is:
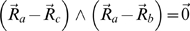
which writes
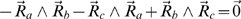
or
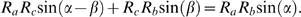



This gives
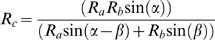
or similarly
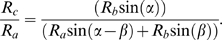



Formula of figure 8D can be derived in the same way.

## Supporting Information

File S1 Data & Software
(2.38 MB ZIP)
