# Supplementary material for: A Global Regulation Inducing the Shape of Growing Folded Leaves
Source: PLoS One. 2009 Nov 23;4(11):e7968. doi: 10.1371/journal.pone.0007968 (PMC2776983; doi:10.1371/journal.pone.0007968)
Supplement: File S1 — Data & Software (2.38 MB ZIP) [file pone.0007968.s001.zip › Supporting Information/figure 11 - data/manual.rtf]

ManualOpen matlab.Choose as "Current directory" the directory which contains this manual. Type "load('Data figure 11/data.mat')" on the command window.To plot the curve for the sinus :Type "prevision_pointe_pointe_art(mat_tout_ordonne,0,1)"To plot the curve for the lobe :Type "prevision_pointe_pointe_art(mat_tout_ordonne,1,1)"
