# Supplementary material for: A Global Regulation Inducing the Shape of Growing Folded Leaves
Source: PLoS One. 2009 Nov 23;4(11):e7968. doi: 10.1371/journal.pone.0007968 (PMC2776983; doi:10.1371/journal.pone.0007968)
Supplement: File S1 — Data & Software (2.38 MB ZIP) [file pone.0007968.s001.zip › Supporting Information/to read first.rtf]

ContentThis folder contains the data corresponding to the figures of the article,as well as software to produce them and the software to fold back leaves(Folding), in Matlab.Manuals are contained in each corresponding folder.
