# Supplementary material for: A Global Regulation Inducing the Shape of Growing Folded Leaves
Source: PLoS One. 2009 Nov 23;4(11):e7968. doi: 10.1371/journal.pone.0007968 (PMC2776983; doi:10.1371/journal.pone.0007968)
Supplement: File S1 — Data & Software (2.38 MB ZIP) [file pone.0007968.s001.zip › Supporting Information/figure 8 and 10 - data /manual.rtf]

ManualThe folder "data figure 8" contains the data measured from folds of sycamore leaves for Figure 8.The folder "data figure 10" contains the data measured from folds of phylodendron  for Figure 10.The data are the coordinates (on the original picture) of the position of the petiole and all the successive main peaks and sinuses of the contour. From this the lengths and angles of veins and anti-veins (all starting from the petiole) are computed.To get Figure 8:Open matlab.Choose as "Current directory" the directory which contains this manual. Type " run tout_f8.m " on the Command window. It extracts the angle and the length of lobes and sinus.Type " prevision(mat_tout_ordonne,1) " for prevision of lobes.Type " prevision(mat_tout_gauche_droite,0) " for prevision of sinus.To get Figure 10:Open matlab.Choose as "Current directory" the directory which contains this manual. Type " run tout_f10.m " on the Command window. It extracts the angle and the length of lobes and sinus.Type " prevision(mat_tout_ordonne,1) " for prevision of lobes.Type " prevision(mat_tout_gauche_droite,0) " for prevision of sinus.
