# Supplementary material for: A Global Regulation Inducing the Shape of Growing Folded Leaves
Source: PLoS One. 2009 Nov 23;4(11):e7968. doi: 10.1371/journal.pone.0007968 (PMC2776983; doi:10.1371/journal.pone.0007968)
Supplement: File S1 — Data & Software (2.38 MB ZIP) [file pone.0007968.s001.zip › Supporting Information/figure 6 and 9 - Folding and data/manual-Folding.rtf]

Folding ManualWith an example fileOpen matlab.Choose as "Current directory" the directory which contains this manual. Choose an example file in the folder "example" for instance "Acer pseudoplatanus".Type "load('example/Acer pseudoplatanus.mat')". It loads the measurement of the veins.Type "plie_N_lobes(gauche,creux_gauche,droit,creux_droit,nervure,creux,contour)" on the Command window.If the leaf has no secondary lobes (for instance Sida hermaphrodita), You have to type: "pli_principaux(nervure,creux,contour)". The leaf is now folded.Do your own foldingOpen matlab.Choose as "Current directory" the directory which contains this manual. Open an image of a leaf of your choice in matlab.Type "run prog_nervure_seul" to record the vein ( "nervure" in the programme) , and the antivein  ("creux" in the programme) of your leaf.The software wait for you to "click" on the picture, recording successively the position of the veinsand anti-veins, starting from the left ending on the right of the leaf, the veins and anti-veins one after the other(first the petiole insertion, then the successive main peaks and valleys of the contour).The secondary veins (and anti-veins) are drawn just after drawing the vein (summit) from which they branch out(from bottom to top). ( in the dialog window "Principale" means "main" and "secondaire" means "secondary").Make the contour of the leaf (with image J for instance: contrast the leaf, selects its border with "magic wand").Record the result ("saveas XY coordinates") into as a "contour.txt" file.Load the result in matlab.Then fold the contour with the above programs : with secondary folds, type "plie_N_lobes(gauche,creux_gauche,droit,creux_droit,nervure,creux,contour)" on the Command window.without secondary folds, type "pli_principaux(nervure,creux,contour)" on the Command window.
