# Supplementary figures and images for: A Global Regulation Inducing the Shape of Growing Folded Leaves
Source: PLoS One. 2009 Nov 23;4(11):e7968. doi: 10.1371/journal.pone.0007968 (PMC2776983; doi:10.1371/journal.pone.0007968)

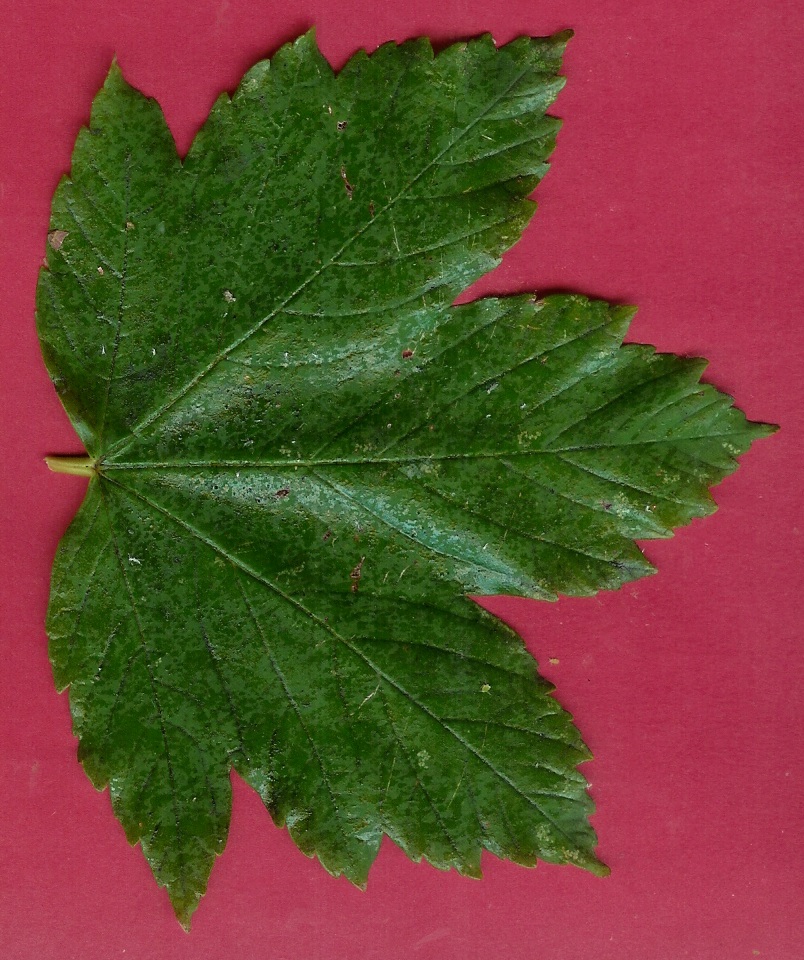

Supplement: File S1 — Data & Software (2.38 MB ZIP) [file pone.0007968.s001.zip › Supporting Information/figure 6 and 9 - Folding and data/example/image/Acer pseudoplatanus.jpg]

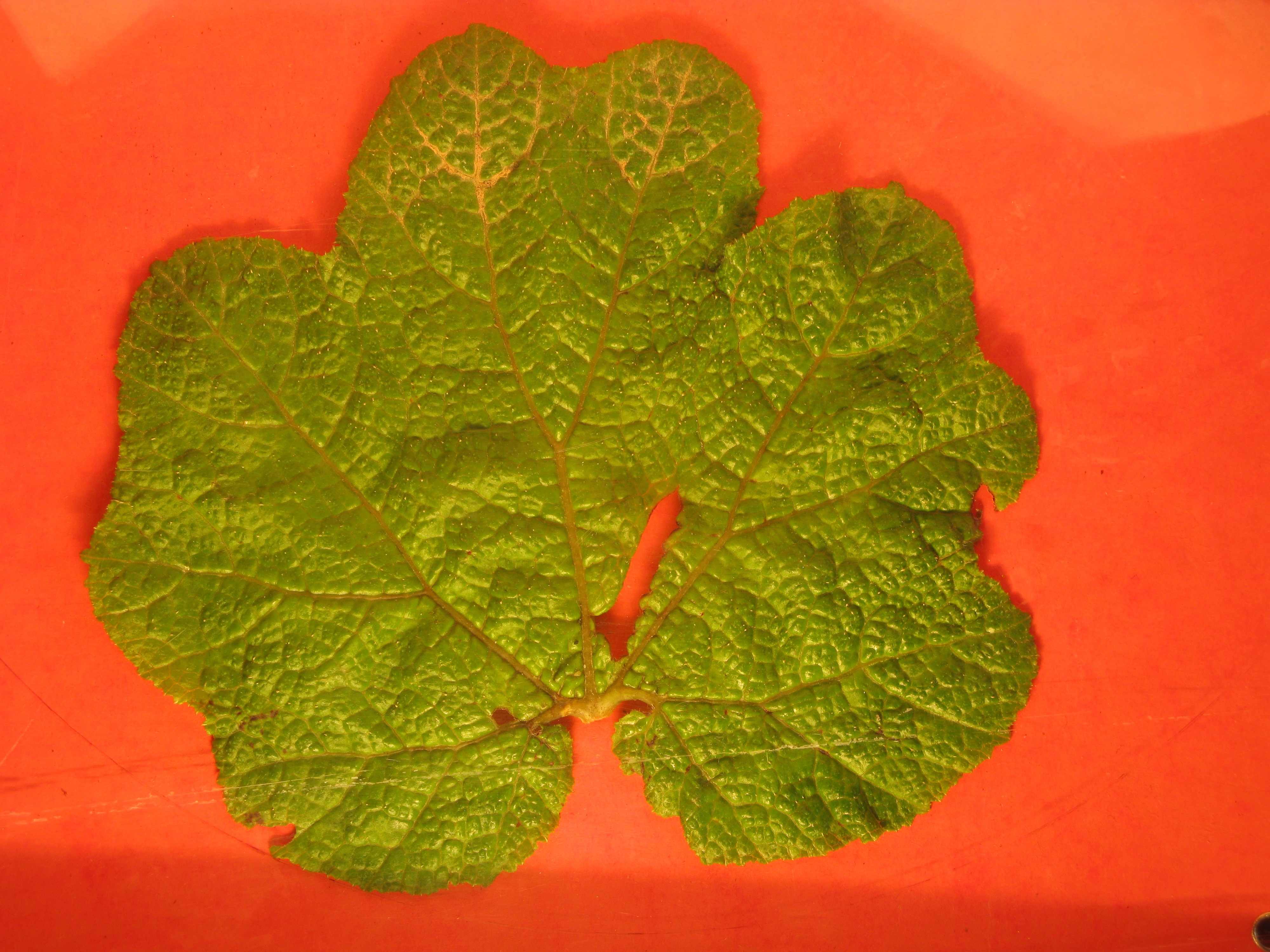

Supplement: File S1 — Data & Software (2.38 MB ZIP) [file pone.0007968.s001.zip › Supporting Information/figure 6 and 9 - Folding and data/example/image/Gunera manicata.jpg]

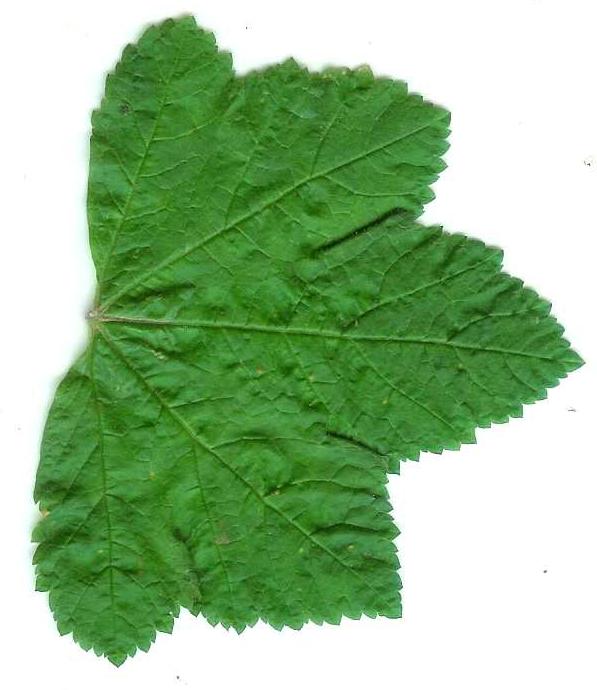

Supplement: File S1 — Data & Software (2.38 MB ZIP) [file pone.0007968.s001.zip › Supporting Information/figure 6 and 9 - Folding and data/example/image/Malva sylvatica.jpg]

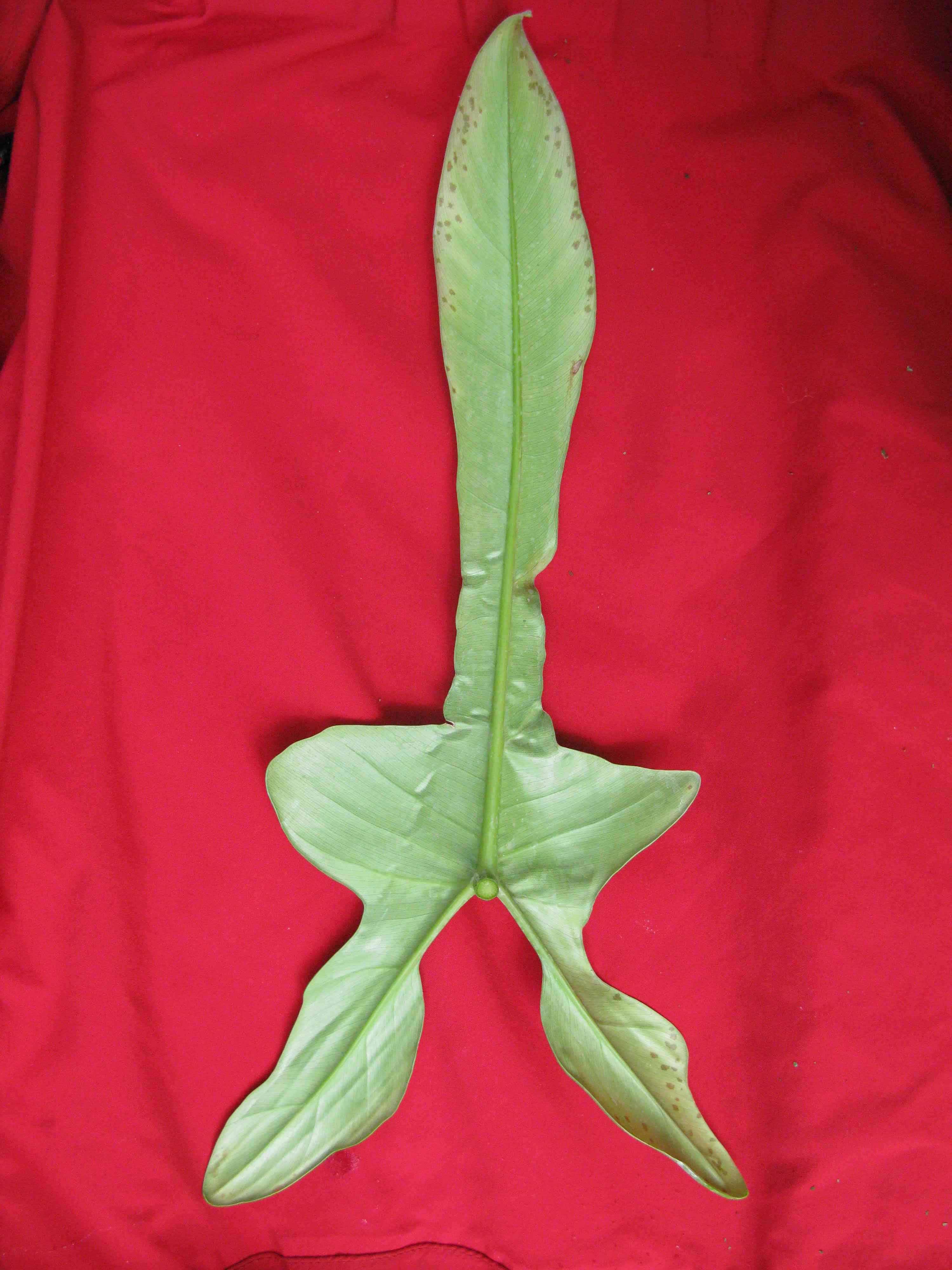

Supplement: File S1 — Data & Software (2.38 MB ZIP) [file pone.0007968.s001.zip › Supporting Information/figure 6 and 9 - Folding and data/example/image/Phylodendron bipenifolium.jpg]

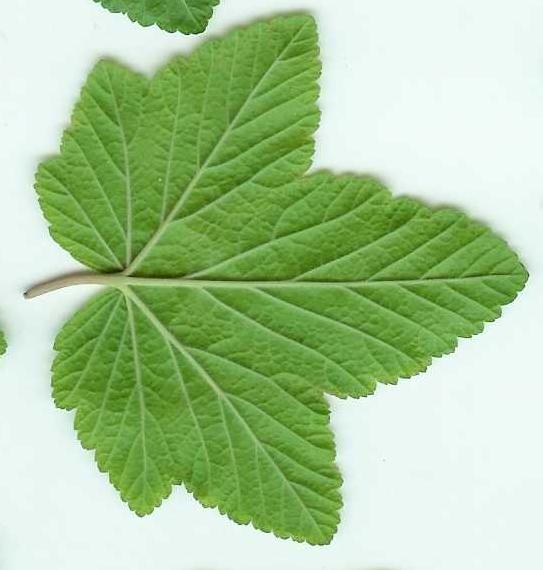

Supplement: File S1 — Data & Software (2.38 MB ZIP) [file pone.0007968.s001.zip › Supporting Information/figure 6 and 9 - Folding and data/example/image/Ribe nigrum.jpg]

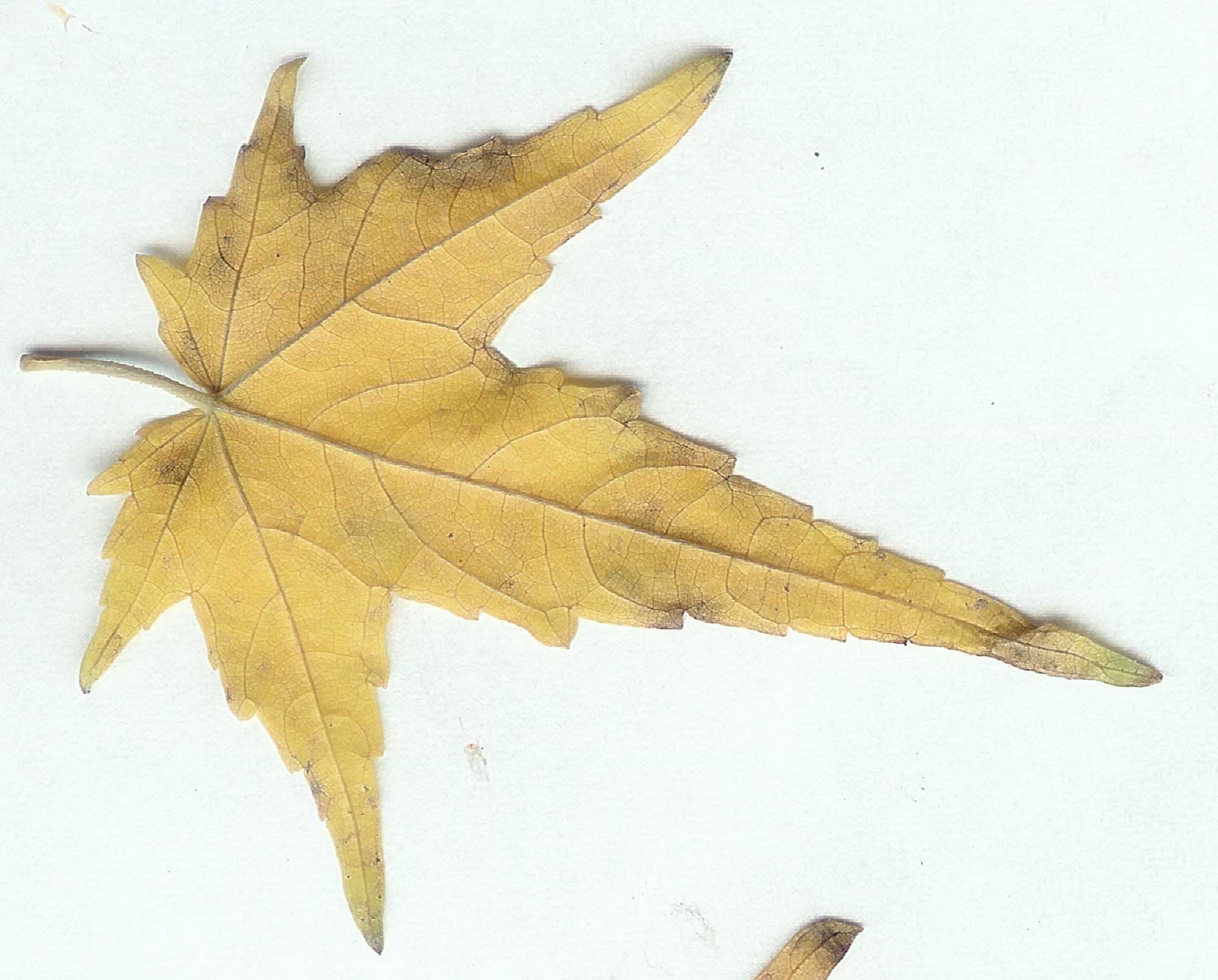

Supplement: File S1 — Data & Software (2.38 MB ZIP) [file pone.0007968.s001.zip › Supporting Information/figure 6 and 9 - Folding and data/example/image/Sida hermaphrodita.jpg]
